# Metal concentrations and KIM-1 levels in school-aged children: a cross-sectional study

**DOI:** 10.1038/s41598-024-62320-8

**Published:** 2024-06-12

**Authors:** Oliver Mendoza‐Cano, Mónica Ríos‐Silva, Irma Gonzalez-Curiel, Arlette A. Camacho-delaCruz, María Fernanda Romo-García, Herguin Benjamin Cuevas-Arellano, Ana Luz Quintanilla‐Montoya, Miguel A. Martínez-Preciado, Pedro Rincón-Avalos, Ángel Gabriel Hilerio-López, Efrén Murillo‐Zamora

**Affiliations:** 1 Facultad de Ingeniería Civil, Universidad de Colima, Carretera Colima-Coquimatlán km 9, Col. Jardines del Llano, 28400 Coquimatlán, México; 2 Facultad de Medicina, Universidad de Colima, Av. Universidad 333, Col. Las Víboras, 28040 Colima, México; 3 Laboratorio de Inmunotoxicología, Unidad Académica de Ciencias Químicas, Universidad Autónoma de Zacatecas, Campus UAZ Siglo XXI, Carretera Zacatecas-Guadalajara KM.6, Col. Ejido La Escondida, 98160 Zacatecas, México; 4 Posdoctorante del Laboratorio de Inmunotoxicología, Unidad Académica de Ciencias Químicas, Universidad Autónoma de Zacatecas, Campus UAZ Siglo XXI, Carretera Zacatecas-Guadalajara KM.6, Col. Ejido La Escondida, 98160 Zacatecas, México; 5 Facultad de Ciencias, Universidad de Colima, Bernal Díaz del Castillo No. 340, Col. Villas San Sebastián, 28045 Colima, México; 6 Comisión Nacional del Agua Dirección Local Colima, Avenida Carlos de La Madrid Béjar S/N, Col. Centro, 28000 Colima, México; 7 Facultad de Enfermería, Universidad de Colima, Avenida Universidad 333, Col. Las Víboras, 28040 Colima, México; 8 Unidad de Investigación en Epidemiología Clínica, Av. Lapislázuli 250, Col. El Haya, 28984 Villa de Álvarez, México

**Keywords:** Child, Environmental pollutants, Kidney, Mexico, Environmental impact, Biomarkers, Diseases

## Abstract

Environmental exposure to heavy metals and metalloids, originating from sources such as mining and manufacturing activities, has been linked to adverse renal effects. This cross-sectional study assessed children's exposure to these elements and its association with urinary kidney injury molecule-1 (KIM-1). We analyzed data from 99 school-aged children residing in nine localities within the state of Colima, Mexico, during the latter half of 2023. Levels of 23 metals/metalloids and urinary KIM-1 were measured using inductively coupled plasma mass spectrometry (ICP-MS) and enzyme-linked immunosorbent assay, respectively. Detectable levels of these contaminants were found in over 91% of participants, with varied exposure profiles observed across locations ($p$ = 0.019). After adjusting for confounding factors like gender, age, and locality, higher levels of six metals/metalloids (boron, cadmium, cesium, lithium, selenium, zinc) were significantly associated with increased KIM-1 levels. Tailored mitigation efforts are crucial to protect children from regional pollutant burdens. However, limitations exist, as our study did not capture all potential factors influencing heavy metal/metalloid and KIM-1 levels.

## Introduction

Exposure to environmental contaminants can have profound implications for the renal health of children^1^. Numerous studies have underscored the potential adverse effects of pollutants on these vital organ systems during childhood^2^.

Despite being one of the smallest states in the country, Colima boasts a variety of economic activities, including sugarcane production in the locality of Quesería, large—scale agriculture in Tecomán and Armería, cement production in Caleras, mining in Minatitlán and Paticajo, major port-related activities in Manzanillo, and thermal power generation in Campos. These diverse industries inherently possess the potential to generate toxic substances, such as heavy metals and metalloids, for the humans and the other ecosystem components^3,4^.

Other sources of exposure beyond the environment are also present. Work environments, such as manufacturing industries, can result in exposure to heavy metals^5^. Moreover, consumer goods like cosmetics and toys may also contain them^6,7^. These non-environmental avenues underscore the significance of monitoring and regulating heavy metal exposure to safeguard human health.

All metals may be toxic depending on several factors, such as their chemical form and concentration, route and duration of exposure, and individual susceptibility^8^. However, some of them (i.e. iron, zinc, copper, and others) are essential for various physiological functions in humans^9^.

Kidneys are particularly sensitive to heavy metals and metalloids^10,11^. This sensitivity arises from divalent metal transporter-1 (DMT-1) expressed in renal tissue^12^. DMT-1 exhibits a high affinity for divalent metals, which are reabsorbed in the proximal tubule and can persist in kidney tissue for up to a decade^13^. The resulting impact on glomerular filtration and tubular function may contribute to long-term renal complications. Kidney Injury Molecule-1 (KIM-1) is a glycoprotein expressed by injured proximal tubular epithelial cells in the kidneys^14^. Its presence in urine, KIM-1, serves as a potential biomarker of kidney injury^15^.

The aim of this study was to assess the impact of environmental pollutants, specifically heavy metals and metalloids, on the kidney function of school-aged children residing in Colima state, Mexico. We sought to describe the children's clinical and paraclinical profiles and evaluate the relationship between heavy metal/metalloid levels and KIM-1 levels. Understanding the potential health implications of exposure to these pollutants is crucial for developing targeted interventions and public health strategies to protect children's renal health.

## Methods

### Modeling the dispersion of contaminants

We accessed data from the Pollutant Release and Transfer Register (RETC) to investigate the dispersion of metals and metalloids into the atmosphere, soil, surface water bodies, and underground water wells spanning 17 years (2004–2021). RETC, a governmental environmental policy mechanism, releases comprehensive data on emissions and movements of contaminants as stipulated by current laws^16^. ^16^Leveraging this dataset, we constructed models to simulate the spread of these pollutants, including the heavy metals and metalloids of interest, in the environment.

**Figure 1:**
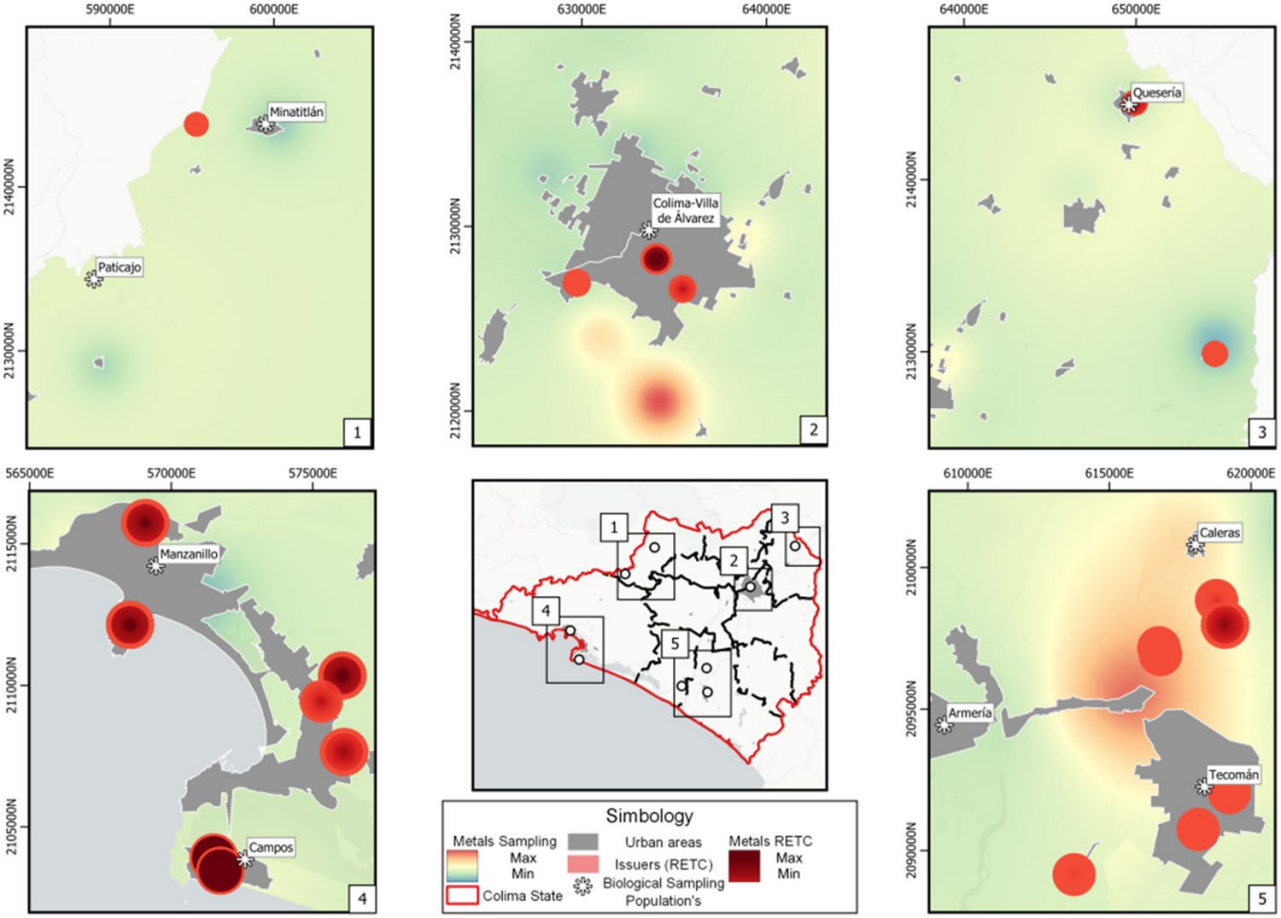
Interpolated map of metalloid concentrations, Mexico 2023.

To process the spatial data and perform interpolation necessary for projecting pollutant concentrations onto the surfaces of the study areas, we employed the QGIS Geographic Information System 3.22 (Open Source Geospatial Foundation Project). QGIS facilitated the manipulation of vectorial information and allowed us to utilize Kriging interpolation to estimate values at unsampled locations. By integrating RETC data and QGIS, we were able to generate detailed spatial representations of metal and metalloid concentrations, aiding in the characterization of environmental contamination in the evaluated localities (Fig. 1).

The model results indicated that metal pollutants released from point sources, such as the RETC issuers, into the surrounding environment over nearly two decades had reached populated localities, including those from which children for the biological sampling were recruited (circles in Fig. 1). However, environmental sampling and chemical analysis conducted in various locations within the study area in 2023 revealed that the pollutants had reached even further areas than those predicted by the modeling approach (blue-green–red faded colored areas also in Fig. 1), and at concentrations significantly higher (by one order of magnitude) than the estimated values from the modeled RETC data. This approach confirmed the fate and transport of the studied pollutants, helping to determine potential dispersion areas from known sources, and also provided a scenario depicting contributions from other sources associated with economic and industrial activities in the region.

### Study design

A cross-sectional epidemiological study was conducted in the second half of 2023, focusing on nine urban and rural areas within the state of Colima, Mexico. The selection of these specific locales was informed by their pre-existing classification as high-risk environmental and health zones. This classification was primarily based on the prevalence of industrial, energy-related, or agricultural activities in these regions, indicating increased exposure to environmental contaminants. Additionally, this classification was corroborated by reports from the RETC and the dispersion models (Fig. 1). The following locations were selected: the metropolitan area of Colima and Villa de Álvarez (the capital of the state; COL), Tecomán (TEC), Caleras (CAL), Armería (ARM), Manzanillo (MAN), Campos (CAM), Quesería (QUE), Paticajo (PAT), and Minatitlán (MIN).

### Study population

This study included children aged 5–12 years old residing in any of the participating localities for at least five years, as reported by their accompanying parent or legal guardian. Children with self-reported chronic non-communicable diseases, those experiencing chronic illnesses at the time of evaluation, and those who had consumed processed foods within 24 h prior to urine sample collection were excluded. The latter exclusion criterion was relevant because processed foods may contain contaminants, such as heavy metals^17^, that could artificially elevate urinary levels. Additionally, these foods may contain additives or preservatives that could potentially increase KIM-1 levels^18^. Participation required informed consent from the parent or legal guardian and assent from the child.

A non-probabilistic, multistage approach was employed for participant selection. In the first stage, elementary schools within the high environmental risk area were identified. The second stage involved identifying eligible children attending these schools. A non-probabilistic, multistage approach was used for participant selection. In the initial stage, elementary schools located in the high environmental risk area were identified. The subsequent stage involved the identification of eligible children attending these schools. Finally, parents or legal guardians of eligible children were invited to an informative workshop through written invitations.

The workshop included a detailed explanation of the study. All children attending the workshops accompanied by a parent or legal guardian, meeting the eligibility criteria, were enrolled. The response rate was 77% and remained consistent across localities. Seventeen children were excluded in total, with 15 out of 17 (92%) excluded due to consuming processed food within 24 h preceding the workshop and urine sample collection. The study sample was integrated by 225 minors.

To assess the heavy metals/metalloids of interest, a stratified random sampling procedure was employed. Children were stratified by residential locality, and a weighted random sample of 99 children was selected, proportional to the size of each stratum. The number of children in whom the biomarkers of interest were assessed was determined entirely by the budget; it represents the maximum number of minors that could be evaluated based on the total funding allocated to the research project for this purpose.

### Data collection

Hereditary and familial backgrounds, along with personal medical history, were assessed using a structured questionnaire. Clinical evaluation focused on chronic respiratory diseases (e.g., asthma), neurological conditions (e.g., stroke, migraine, epilepsy), immunological/allergic disorders (e.g., atopic dermatitis, allergies), and psychiatric conditions (e.g., depression, anxiety). In most cases (60%), mothers served as the primary informants.

Anthropometric measurements were obtained. To evaluate the nutritional status of the participants, Z scores for height-for-age, weight-for-age, and body mass index (BMI) for age were computed. These Z scores were calculated using the normative Mexican standards (height-for-age, and weight-for-age)^19^ and the WHO Child Growth Standards (BMI-for-age)^20^.

### Sample collection

Blood samples (4 mL) were collected by peripheral venipuncture into sterile tubes without additives. Following collection, they were maintained at 4 °C until centrifugation for 10 min at 1000 × g. Fresh serum was then used for creatinine determination.

Morning spot urine samples were collected via spontaneous micturition in sterile containers (150 mL) and maintained at 4 °C. These samples were used for the quantification of specific gravity, urea, urea nitrogen, and creatinine. Serum creatinine was used to calculate the glomerular filtration rate (GFR) for each participant using the adapted Schwartz formula for pediatric patients. The remaining urine was aliquoted for the determination of KIM-1 levels and exposure biomarkers.

The rationale for selecting KIM-1 stemmed from two main considerations. KIM-1 has been recognized for its sensitivity and specificity in detecting renal tubular injury, particularly in cases of acute kidney injury^21^. Moreover, its levels have demonstrated a correlation with the extent of renal damage and have exhibited predictive potential for adverse outcomes^22^. Given its capacity to identify early signs of renal impairment and its association with clinical prognosis, KIM-1 is a valuable tool for evaluating kidney injury in pediatric patients.

### Laboratory methods for KIM-1 and heavy metals and metalloids quantification

Urine KIM-1 was quantified using a commercial ELISA kit (ab235081, ABCAM). Briefly, 50 μL aliquots were added to wells and shaken for 30 min. The plate was then washed repeatedly before each subsequent step. Detection antibody conjugate (50 μL) was added, excluding blank and NSB controls, followed by another incubation. TMB substrate (50 μL) was then added and incubated for a set time (refer to manufacturer's instructions). The reaction was stopped with sulfuric acid, and absorbance was measured at 450 nm using a plate reader (FLUOROSKAN, Thermo Fisher). The assay specifications include a recovery percentage range of 89–127% and a limit of detection of 1.279 pg/mL determined by interpolating 2 standard deviations from the mean of 0 pg/mL. Additionally, the cross-reactivity of related compounds is less than 0.02% for TIM-3 and TIM-4.

Urine metal and metalloid levels were determined using Inductively Coupled Plasma Mass Spectrometry (NexION 300D, PerkinElmer), employing a validated method provided by the Research and Service Laboratory in Toxicology at the Center for Research and Advanced Studies of the National Polytechnic Institute (CINVESTAV). All samples were analyzed in duplicate to ensure analytical reliability. As part of the analytical quality control, the accuracy and precision of the determination were assessed, with a criterion that the analytical coefficient of variation did not exceed 10% in the duplicate samples. For assessing accuracy, multi-element urine reference standards (QM-U-Q2104, 105, 106, 113, 114, and 115) from the Institut National de Santé Publique du Québec (INSPQ) were utilized. These standards were analyzed alongside the study samples, achieving an accuracy percentage within the range of 80–120%. Additionally, the results were adjusted for urine specific gravity.

### Statistical analysis

Summary statistics were computed based on sex and location of residence. Chi-squared, t, or U tests, as well as Kruskal–Wallis tests, were employed as appropriate. The significance level ($\alpha$) was set at 5%.

Given that there is no universal consensus on acceptable urinary levels of heavy metals and metalloids in children, we chose to use the 80th percentile as a cut-off. Therefore, children with urinary levels of any given heavy metals or metalloid equal to or above the 80^th^ percentile were considered as having high levels and were compared to those with levels below this cut-off (low-level group).

For crude KIM-1, we calculated the geometric mean (GM) and 95% confidence interval (CI) for its levels. We utilized the previously published mean levels (179 pg/ml) documented in a population of 227 Mexican children as a cut-off^23^. Consequently, the levels were dichotomized as < 179 pg/mL or ≥ 179 pg/mL (high-level group).

Finally, we investigated the association between levels of heavy metals and metalloids, adjusted for specific gravity, and unadjusted KIM-1 levels using generalized linear models (GLMs). We calculated regression coefficients with 95% confidence intervals (CIs) for each metal, building two models per metal. The first model examined the direct relationship between the metal and KIM-1, without accounting for potential confounding variables. The latter one included gender, age, and locality of residence as potential confounders. The potential confounding variables incorporated into the model were identified based on previously published data indicating varied exposure profiles in relation to sex and age^24,25^. The inclusion of residency locality was deemed necessary owing to its industrial and productive activities, which could potentially result in the release of specific heavy metals and metalloids. This approach would allow for a more nuanced understanding of how heavy metals and metalloids may influence KIM-1 levels.

### Ethical considerations

The written informed consent to participate was obtained from each parent or legal guardian of the children, as well as the assent to participate from the children themselves. The study protocol underwent thorough review and received approval from the Committee of Ethics in Health Research of the Ministry of Health in the state where the study was conducted (approval CBCANCL2306023-PRONAII-17).

## Results

A total of 225 school-aged children (115 girls and 110 boys) were recruited, and their mean age (± standard deviation) was 8.2 ± 1.6 years old (Supplementary data 1). No significant differences were found in the gender-stratified mean age ($p$= 0.333) nor in the proportion of girls and boys per locality of residence ($p$= 0.512).

Here we analyze data from the 99 randomly selected children, whose characteristics are summarized in Table 1.. The characteristics of this subset of infants were statistically similar when compared to those of the total sample size (Supplementary data 1).

**Table 1:** Characteristics of the study sample for selected variables, Mexico 2023.

Characteristic		Girls	Boys	*p*
	Overall n= 99	n= 52	n= 47	
Age (mean ± S.D.), years	8.1 ± 1.5	8.0 ± 1.5	8.2 ± 1.5	0.456
Age group (tertiles), years				
5–7	39 (39.3)	23 (44.2)	16 (34.0)	0.553
8–9	42 (42.5)	20 (38.5)	22 (46.8)	
10–12	18 (18.2)	9 (17.3)	9 (19.2)	
Height (mean ± S.D.), cm	130.6 ± 11.0	129.9 ± 11.4	131.2 ± 10.6	0.576
Weight (mean ± S.D.), kg	31.8 ± 11.5	31.3 ± 12.5	32.4 ± 10.4	0.642
Children per locality of residence				
COL	15 (15.2)	10 (19.2)	5 (10.6)	0.686
TEC	9 (9.1)	4 (7.7)	5 (10.6)	
ARM	9 (9.1)	5 (9.6)	4 (8.5)	
CAL	9 (9.1)	4 (7.7)	5 (10.6)	
MAN	11 (11.1)	5 (9.6)	6 (12.8)	
CAM	10 (10.1)	5 (9.6)	5 (10.6)	
QUE	20 (20.2)	8 (15.4)	12 (25.6)	
MIN	4 (4.0)	2 (3.9)	2 (4.3)	
PAT	12 (12.1)	9 (17.3)	3 (6.4)	
Nutritional status:				
Weight-for-age				
Malnutrition (mild—moderate)	12 (12.1)	8 (15.4)	4 (8.5)	0.503
Normal	55 (55.6)	29 (55.8)	26 (55.3)	
Overweight—Obesity	32 (32.3)	15 (28.8)	17 (36.2)	
Height-for-age				
Low	11 (11.1)	3 (5.8)	7 (14.9)	0.319
Normal	73 (73.7)	41 (78.8)	33 (70.2)	
High	15 (15.2)	8 (15.4)	7 (14.9)	
BMI-for-age				
Low	2 (2.2)	2 (4.4)	0 (0)	0.601
Normal	60 (60.4)	33 (63.0)	27 (57.8)	
Overweight—Obesity	37 (37.4)	17 (32.6)	20 (42.2)	
Ever medical diagnosis of (any, yes)				
Chronic respiratory disease	5 (5.1)	1 (1.9)	4 (8.5)	0.135
Neurological disease	6 (6.1)	3 (5.8)	3 (6.4)	0.898
Immunological or allergic disease	10 (10.1)	8 (15.4)	2 (4.3)	0.067
Psychiatric disease	7 (7.1)	3 (5.8)	4 (8.5)	0.595

SD, Standard Deviation; BMI, Body Mass Index; COL, Colima – Villa de Álvarez; TEC, Tecomán; ARM, Armería; CAL, Caleras; MAN, Manzanillo; CAM, Campos; QUE, Quesería; MIN, Minatitlán; PAT, Paticajo.

1) The absolute (n) and relative (%) frequencies are presented, unless the arithmetic mean and SD are specified; 2) The* p* values from chi-squared or t tests are presented accordingly; 3) The nutritional status was evaluated using Z scores determined with the normative Mexican standards, and the WHO Child Growth Standards, as corresponding: Weight-for-age (Low, ≤ -3 to < -1; Normal, -1 to +1; High, > +1); Height-for-age (Low, ≤ -2 to < -1; Normal, -1 to +1; High, > +1); BMI-for-age (Low, < -2; Normal, ≥ 2 to < +2; High, ≥ +3).

As presented in Table 1., most of the analyzed girls and boys exhibited a normal nutritional status, and no significant gender-related differences were observed. The nutritional status was also similar among the analyzed localities in terms of weight-for-age, height-for-age, and BMI-for-age.

### Exposure biomarkers

#### Heavy metals and metalloids

Detectable levels of the analyzed heavy metals and metalloids were found in more than 91% ($n$ = 90/99) of participants. However, the percentage was lower for manganese, which was detectable (according to the limit of detection [LOD] ≤ 0.065 ng/ml) in 52.5% ($n$ = 52/99) of children.

The remaining proportions of participants with detectable levels of these substances were as follows: aluminum (LOD ≤ 0.045 ng/ml), 96.0% ($n$= 95/99); arsenic (LOD ≤ 0.041 ng/ml), 93.9% ($n$= 93/99); barium, 100%; boron, 100%; cadmium, 100%; cesium, 100%; cobalt (≤ 0.053 ng/ml), 94.9% ($n$= 94/99); copper (≤ 0.112 ng/ml), 93.9% ($n$= 93/99); iodine, 100%; iron, 100%; lead, 100%; lithium, 100%; mercury (LOD ≤ 0.079 ng/ml), 99.0% ($n$= 98/99); molybdenum, 100%; nickel (LOD ≤ 0.043 ng/ml), 98.0% ($n$= 97/99); selenium, 100%; strontium (LOD ≤ 0.529 ng/ml), 99.0% ($n$= 98/99); tellurium, 100%; thallium (LOD ≤ 0.063 ng/ml), 91.9% ($n$= 91/99); tin (LOD ≤ 0.131 ng/ml), 98.0% ($n$= 97/99); titanium (LOD ≤ 0.174 ng/ml), 98.0% ($n$= 97/99); zinc, 100%. Urinary levels of the analyzed xenobiotics below the LOD were considered undetectable for the purposes of our study.

**Figure 2:**
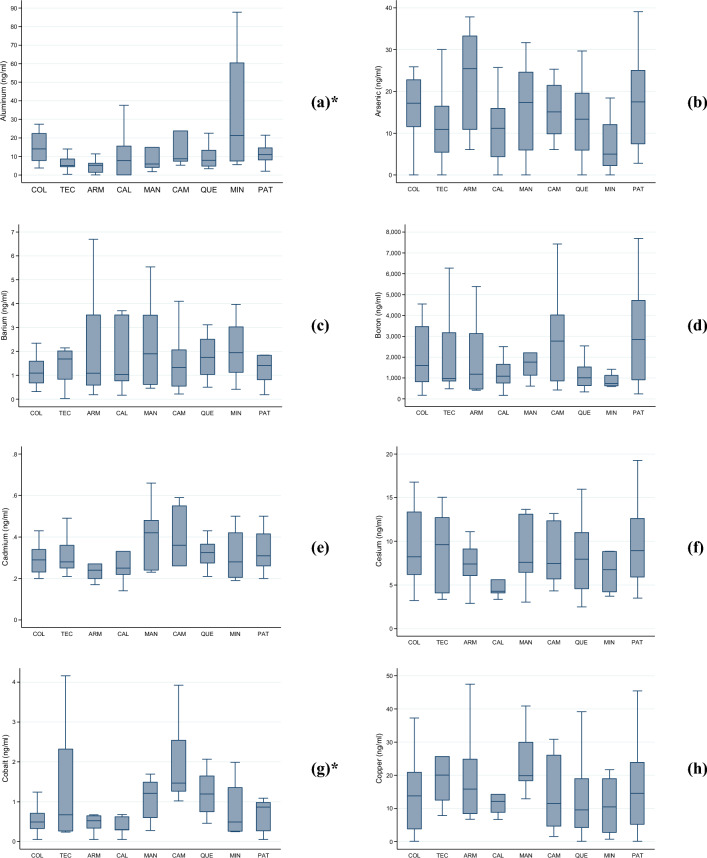

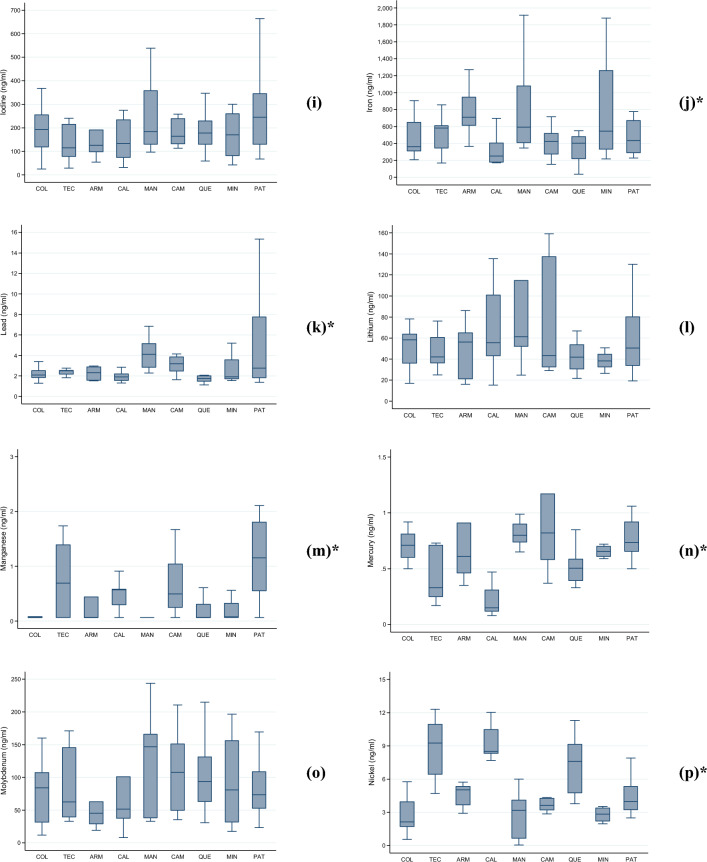

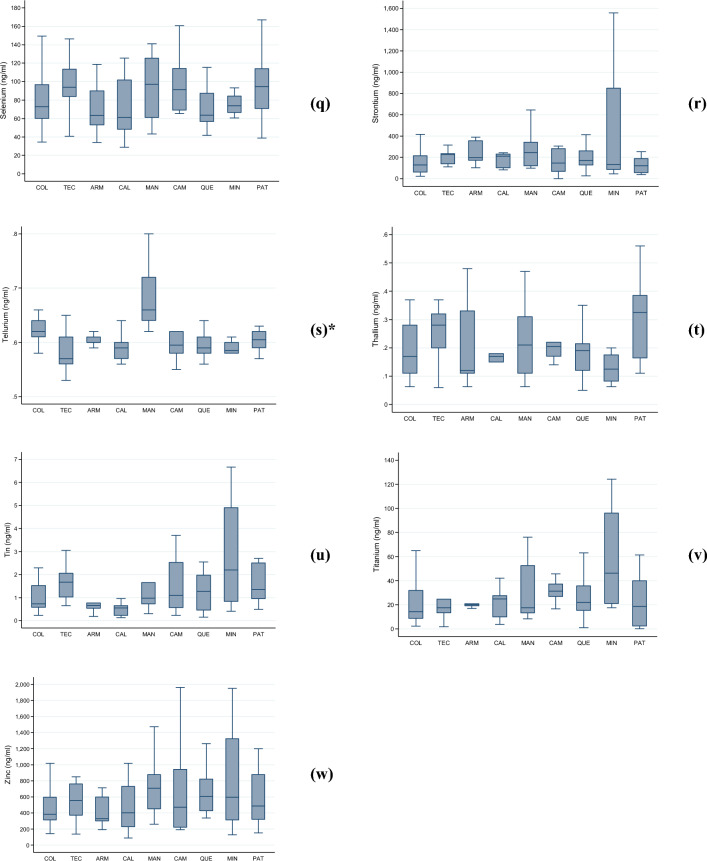
(**a**–**w**). Levels (median, interquartile range, and total range) of twenty-three heavy metals and metalloids (ng/ml) in school-aged children analyzed according to their locality of residence, Mexico 2023. The following were analyzed: (**a**) aluminum; (**b**) arsenic; (**c**) barium; (**d**) boron; (**e**) cadmium; (**f**) cesium; (**g**) cobalt; (**h**) copper; (**i**) iodine; (**j**) iron; (**k**) lead; (**l**) lithium; (**m**) manganese; (**n**) mercury; (**o**) molybdenum; (**p**) nickel; (**q**) selenium; (**r**) strontium; (**s**) tellurium; (**t**) thallium; (**u**) tin; (**v**) titanium; and (**w**) zinc. Abbreviations: COL, Colima—Villa de Álvarez; TEC, Tecomán; ARM, Armería; CAL, Caleras; MAN, Manzanillo; CAM, Campos; QUE, Quesería; MIN, Minatitlán; PAT, Paticajo. * Kruskal–Wallis equality-of-populations rank test, $p$ < 0.05.

Figure 2a–w presents the median levels (along with the IQR and total range) of the assessed heavy metals and metalloids in our randomly selected subset of children. We observed significant differences in median levels across localities of residence for eight of the evaluated metals: aluminum ($p$ = 0.042), cobalt ($p$ < 0.001), iron ($p$ = 0.001), manganese ($p$ < 0.001), mercury ($p$ < 0.001), nickel ($p$ < 0.001), lead ($p$ < 0.001), and tellurium ($p$ < 0.001).

As presented in Table 2, when the urinary levels of heavy metals and metalloids were dichotomized based on the 80th percentile, diverse profiles were observed. Significant disparities were noted for cobalt, iron, lead, manganese, mercury, nickel, tellurium, and thallium.

**Table 2:** Prevalence of urinary levels of analyzed heavy metals and metalloid equal to or above the 80th percentile in school-aged children, Mexico 2023. Significant values are in bold.

	COL	TEC	ARM	CAL	MAN	CAM	QUE	MIN	PAT	$p$
	$n$= 15	$n$= 9	$n$= 9	$n$= 9	$n$= 11	$n$= 10	$n$= 20	$n$= 4	$n$= 12	
Aluminum	46.7	0	11.1	33.3	27.3	40.0	25.0	50.0	33.3	0.334
Arsenic	33.3	11.1	55.6	22.2	27.3	30.0	20.0	0	50.0	0.282
Barium	20.0	11.1	44.4	33.3	45.5	20.0	40.0	25.0	16.7	0.537
Boron	33.3	33.3	33.3	11.1	18.2	50.0	20.0	0	50.0	0.310
Cadmium	20.0	22.2	22.2	22.2	63.6	40.0	20.0	25.0	25.0	0.325
Cesium	40.0	33.3	11.1	22.2	36.4	40.0	25.0	0	33.3	0.725
Cobalt	13.3	33.3	11.1	11.1	45.5	70.0	45.0	25.0	0	**0.006**
Copper	20.0	44.4	33.3	22.2	45.5	40.0	20.0	25.0	25.0	0.776
Iodine	26.7	22.2	22.2	11.1	45.5	30.0	20.0	25.0	58.3	0.337
Iron	26.7	22.2	77.8	11.1	45.5	20.0	10.0	50.0	33.3	**0.020**
Lead	20.0	11.1	22.2	0	72.7	60.0	10.0	25.0	50.0	**0.001**
Lithium	26.7	22.2	44.4	33.3	54.6	30.0	15.0	0	33.3	0.391
Manganese	0	55.6	22.2	55.6	0	40.0	5.0	25.0	75.0	** < 0.001**
Mercury	26.7	0	33.3	0	63.6	60.0	20.0	0	33.3	**0.006**
Molybdenum	13.3	33.3	22.2	22.2	63.6	40.0	25.0	25.0	25.0	0.303
Nickel	0	66.7	11.1	100	0	10.0	55.0	0	8.3	** < 0.001**
Selenium	20.0	44.4	22.2	22.2	45.5	40.0	25.0	0	33.3	0.655
Strontium	20.0	22.2	44.4	22.2	54.6	30.0	30.0	25.0	16.7	0.601
Tellurium	40.0	11.1	11.1	22.2	90.9	0	20.0	0	8.3	** < 0.001**
Thallium	26.7	55.6	33.3	11.1	36.4	20.0	10.0	0	66.7	**0.017**
Tin	20.0	55.6	22.2	0	27.3	30.0	30.0	50.0	41.7	0.317
Titanium	20.0	22.2	22.2	22.2	27.3	50.0	30.0	50.0	33.3	0.829
Zinc	20.0	33.3	11.1	33.3	45.5	30.0	30.0	25.0	33.3	0.881

COL, Colima – Villa de Álvarez; TEC, Tecomán; ARM, Armería; CAL, Caleras; MAN, Manzanillo; CAM, Campos; QUE, Quesería; MIN, Minatitlán; PAT, Paticajo.

Cut-offs (ng/ml): Aluminum (≥ 14.2), Arsenic (≥ 20.7), Barium (≥ 2.2), Boron (≥ 2,543.4), Cadmium (≥ 0.40), Cesium (≥ 11.2), Cobalt (≥ 1.3), Copper (≥ 21.3), Iodine (≥ 239.4), Iron (≥ 611.0), Lead (≥ 3.0), Lithium (≥ 61.2), Manganese (≥ 0.6), Mercury (≥ 0.8), Molybdenum (≥ 130.8), Nickel (≥ 7.0), Selenium (≥ 103.1), Strontium (≥ 240.6), Tellurium (≥ 0.63), Thallium (≥ 0.3), Tin (≥ 1.54), Titanium (≥ 32.1), and Zinc (≥ 723.8).

1) The relative (%) frequen**c**ies are presented, along with the $p$-value from the chi-squared test; 2) The levels of the twenty-three analyzed heavy metals and metalloids were adjusted for urine density.

In CAM, children appeared to be exposed to elevated levels of cobalt, with 70% ($n$ = 7/10) of them exhibiting levels ≥ 1.3 ng/mL. In ARM, nearly 78% ($n$ = 7/9) of the examined girls and boys had iron levels ≥ 611.0 ng/mL. The highest lead exposures were documented in MAN, and CAM, both geographically proximate, where 72.7% ($n$ = 8/11) and 60.0% ($n$ = 6/10) of children exhibited lead levels equal to or exceeding 3.0 ng/mL.

PAT displayed a prevalence of 75% ($n$ = 9/12) of children with manganese levels ≥ 0.6 ng/mL. MAN and CAM exhibited the highest mercury levels (63.6% [$n$ = 7/11] and 60.0% [$n$ = 6/10] with levels ≥ 0.8 ng/mL). All children in CAL had nickel levels ≥ 7.0 ng/mL. Tellurium exposure appeared particularly significant in MAN, where nearly 91% ($n$ = 10/11) of children had levels ≥ 0.63 ng/mL. Lastly, the highest thallium levels were observed in PAT, with 66.7% ($n$ = 8/12) of children having levels ≥ 0.3 ng/mL.

### Effect biomarkers

#### Glomerular filtration rate

The GFR was within the normal range (> 90 mL/min/1.73 m^2^)^26^ for all the children included in the analysis, and no statistically significant gender-related differences were observed ($p$= 0.300). However, significant ($p$= 0.022) variations in median rates were noted when analyzing data based on the locality of residence. In descending order, the median GFR values (mL/min/1.73 m^2^) for each locality were as follows: COL (179.3, IQR 152.2–187.7); CAM (162.9, IQR 146.7–169.6); MAN (160.8, IQR 145.3–187.2); QUE (159.8, IQR 137.8–176.5); TEC (156.9, IQR 136.0–166.5); MIN (152.9, IQR 150.3–171.6); ARM (150.6, IQR 138.2–171.1); CAL (143.8, IQR 127.6–162.8); and PAT (143.2, IQR 132.8–169.9).

#### KIM-1

**Figure 3:**
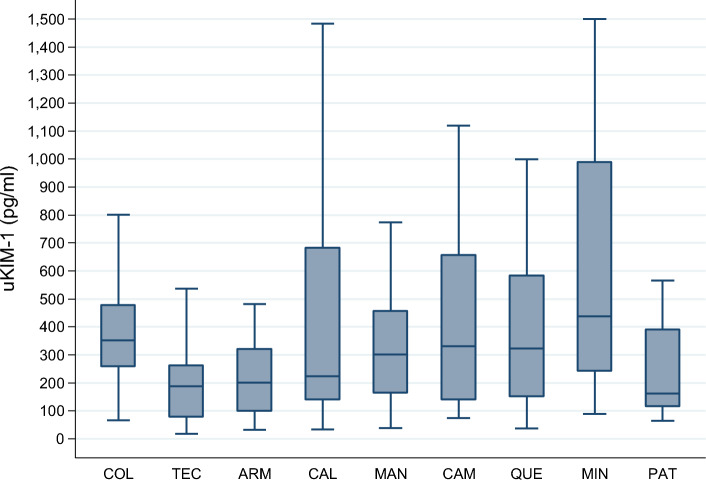
Levels (median, interquartile range, and total range) of the urinary kidney injury molecule-1 (KIM-1) in the analyzed school-aged children by locality of residence, Mexico 2023. COL, Colima—Villa de Álvarez; TEC, Tecomán; ARM, Armería; CAL, Caleras; MAN, Manzanillo; CAM, Campos; QUE, Quesería; MIN, Minatitlán; PAT, Paticajo. Kruskal–Wallis equality-of-populations rank test, $p$ = 0.019.

The total median KIM-1 level was 269.8 pg/mL (IQR 141.6–431.0), and it was similar ($p$ = 0.408) between girls (261.8 pg/ml, IQR 131.9–393.9) and boys (304.6 pg/ml, IQR 152.6–479.6). As observed in Fig. 3, significant ($p$ = 0.019) differences were observed in the levels within the localities of residence.

The GM of KIM-1 levels, used for comparing the observed levels with the applied cut-off and in descending order, were as follows: CAM, 401.3 pg/mL (95% CI 189.2–851.0); MIN, 399.8 pg/mL (95% CI 63.4–2,521.6); COL, 280.7 pg/mL (95% CI 192.7–409.0); CAL, 277.0 pg/mL (95% CI 116.7–657.4); QUE, 232.1 pg/mL (95% CI 154.5–348.6); MAN, 267.3 pg/mL (95% CI 144.2–495.4); ARM, 213.3 pg/mL (95% CI 122.8–370.5); TEC, 195.1 pg/mL (95% CI 98.7–385.6); and PAT, 188.5 pg/mL (95% CI 117.3–302.8).

There were no significant differences in the prevalence of KIM-1 levels ≥ 179 ng/mL among girls and boys ($p$ = 0.364), age groups ($p$ = 0.965), or residential localities ($p$ = 0.562). The overall estimate (of KIM-1 levels ≥ 179 ng/mL) in the study sample was 65.7% ($n$ = 65/99).

### Association analysis

Table 3 displays the regression coefficients for the associations between KIM-1 and various metals/metalloids. Adjusting for age, gender, and locality of residence as potential confounders generally yielded similar coefficients compared to the unadjusted analysis. However, some notable differences emerged. Eight significant associations were identified in the adjusted models.

**Table 3:** Association between KIM-1 Levels and heavy metals and metalloids in school-aged children, Mexico 2023.

	$\beta$(95% CI),$p$	
	Bivariate analysis	Multiple analysis
Aluminum	1.78 (− 0.99 to 4.55), 0.208	1.96 (− 0.57 to 4.48), 0.129
Arsenic	3.56 (− 4.08 to 11.21), 0.361	3.49 (− 3.61 to 10.59), 0.335
Barium	− 3.73 (− 5.56 to − 1.91), < 0.001	− 3.19 (− 5.03 to − 1.34), 0.001
Boron	0.04 (0.01 to 0.08), 0.039	0.04 (0.01 to 0.08), 0.038
Cadmium	866.6 (38.2 to 1,695.0), 0.040	818.21 (49.35 to 1,587.08), 0.037
Cesium	22.74 (3.44 to 42.05), 0.021	20.92 (3.01 to 38.83), 0.022
Cobalt	124.86 (− 27.11 to 276.82), 0.107	147.73 (− 8.38 to 303.84), 0.064
Copper	5.56 (− 0.31 to 11.43), 0.063	5.49 (− 0.12 to 11.09), 0.055
Iodine	0.19 (− 0.21 to 0.59), 0.352	0.16 (− 0.25 to 0.57), 0.435
Iron	0.10 (− 0.14 to 0.35), 0.418	0.09 (− 0.13 to 0.31), 0.434
Lead	28.91 (− 19.69 to 77.52), 0.244	30.38 (− 17.53 to 78.29), 0.214
Lithium	2.75 (1.14 to 4.36), 0.001	2.72 (1.30 to 4.14), < 0.001
Manganese	− 0.18 (− 0.22 to − 0.13), < 0.001	− 0.14 (− 0.19 to − 0.09), < 0.001
Mercury	83.16 (− 18.47 to 184.80), 0.109	95.27 (− 23.44 to 213.98), 0.116
Molybdenum	0.78 (− 0.28 to 1.84), 0.150	0.68 (− 0.36 to 1.73), 0.201
Nickel	2.69 (− 18.67 to 24.05), 0.805	1.56 (− 20.33 to 23.45), 0.889
Selenium	3.93 (1.35 to 6.50), 0.003	3.77 (1.44 to 6.09), 0.001
Strontium	− 0.02 (− 0.22 to 0.18), 0.869	− 0.01 (− 0.20 to 0.17), 0.872
Tellurium	− 2,266.3 (− 4,790.8 to 258.2), 0.078	− 2,306.9 (− 4,737.0 to 123.1), 0.063
Thallium	259.32 (− 325.47 to 844.11), 0.385	171.47 (− 458.63 to 801.56), 0.594
Tin	70.60 (− 29.51 to 170.70), 0.167	68.07 (− 30.00 to 166.13), 0.174
Titanium	2.20 (− 1.54 to 5.95), 0.249	1.69 (− 1.71 to 5.10), 0.330
Zinc	0.32 (0.06 to 0.59), 0.017	0.31 (0.06 to 0.55), 0.015

: KIM-1, urinary kidney injury molecule-1; CI, confidence interval.

Generalized linear models (GLMs) were used to calculate regression coefficients (β) and 95% confidence intervals (CIs); 2) Estimates from the multiple analysis were adjusted for gender, age, and locality of residence.

Boron ($\beta$ = 0.04, 95% CI 0.01–0.08; $p$ = 0.038), cadmium ($\beta$ = 818.21, 95% CI 49.35–1,587.08; $p$ = 0.037), cesium ($\beta$ = 20.92, 95% CI 3.01–38.83; $p$ = 0.022), lithium ($\beta$ = 2.72, 95% CI 1.30–4.14; $p$ < 0.001), selenium ($\beta$ = 3.77, 95% CI 1.44–6.09; $p$ = 0.001), and zinc ($\beta$ = 0.31, 95% CI 0.06–0.55; $p$ = 0.015), all showed positive associations with KIM-1 levels, suggesting potentially increased risk of kidney injury with higher levels of these elements.

Barium ($\beta$ = -3.19, 95% CI − 5.03 to − 1.34; $p$ = 0.001) and manganese ($\beta$ = − 0.14, 95% CI − 0.19 to − 0.09; $p$ < 0.001) displayed negative associations with KIM-1. No other significant associations were observed in the adjusted models.

## Discussion

This study suggests the differential environmental exposure of young girls and boys to heavy metals and metalloids, and their potential influence on kidney function. Children across different locations displayed varying exposure profiles, likely linked, at least in part, to the dominant industrial and productive activities in their respective areas^27^. This suggests that mitigation efforts to protect children's health from these toxic substances must be tailored to the specific pollutant landscape of each region. However, it is noteworthy that, currently, most of the assessed heavy metals and metalloids lack a defined safe level for this specific age group^28^.

It is essential to interpret our findings cautiously, considering the methodological limitations of our cross-sectional study. KIM-1 may be influenced by multiple intrinsic factors, such as hormonal and metabolic status, that were not measured in our study^29^. Additionally, the sample size should be considered when interpreting our results, particularly when assessing statistical differences between the included localities. Despite the sample size per stratum, we evaluated these differences with the aim of providing an initial characterization of the exposure profile per residence site. Conducting measurements of heavy metals and metalloids in a larger number of individuals, particularly in countries with limited funding sources, is often cost-prohibitive.

Kidney function was normal in all examined girls and boys (GFR (> 90 mL/min/1.73 m^2^). The median unadjusted KIM-1 level in our study was 275.0 pg/mL (IQR 146.8–462.2). When considering the geometric mean (GM), the overall estimate for unadjusted KIM-1 was 252.8 pg/mL (95% CI 225.5–283.4). This value represents the typical KIM-1 level in our study population and is approximately 17% lower than the GM reported in school-aged children from Villa de Reyes, Mexico (304.6 pg/ml, 95% CI 216.8–428.1)^30^. This difference suggests potential geographical variations in KIM-1 levels, possibly linked to differences in environmental exposures and raise questions about potential environmental influences on this biomarker.

In our study, the median KIM-1 levels were exhibited a 1.5-fold increase in children residing in MIN (438.0 pg/ml, IQR 243.0–989.8), when compared to the overall estimate. This locality is distinguished by mining activities, primarily focused on the extraction of iron mineral pellets. As presented in Fig. 1j, the infants residing in this locality had the third highest median levels of iron (544.2 ng/ml, IQR 333.0–1,260), just after those from ARM (710.3 ng/ml, IQR 611.0–946.67) and MAN (592.8 ng/ml, IQR 407.5–1,078.0). The environmental exposure to high levels of iron has been associated with cardiovascular diseases^31^.

The use of a cutoff point for KIM-1 in one Mexican population of apparently healthy children^23^ to analyze another Mexican population has methodological implications. Firstly, by employing a specific cutoff point tailored to the population of interest, more precise and relevant results can be obtained, helping to mitigate the risk of extrapolating data from other populations with genetic, environmental, or lifestyle differences that could influence biomarker levels. However, it is important to acknowledge that using a specific cutoff point for one population has limitations, as it may not be applicable to populations with significant differences in relevant characteristics. Additionally, the absence of a universal cutoff point for many biomarkers makes it challenging to directly compare results between different populations or studies.

Our analysis identified a significant positive association between KIM-1 levels and six xenobiotics (boron, cadmium, cesium, lithium, selenium, and zinc). While the mechanisms for all six associations require further investigation, existing literature supports the potential nephrotoxic effects of the first two: boron and cadmium^32,33^.

Boron may potentially produce damage the kidneys through several mechanisms. One major mechanism is oxidative stress, wherein boron can generate reactive oxygen species that damage cells and tissues^34^. Additionally, this xenobiotic can disrupt ion channels and transporters in renal cells, affecting their normal physiological processes and potentially causing kidney damage^35^. The chronic exposure to high levels of cadmium can lead to kidney damage through a combination of direct tubular toxicity, oxidative stress, disruption of renal transport mechanisms, and activation of pro-inflammatory pathways^36,37^.

Among girls and boys analyzed, the highest median levels of boron were observed in CAM (2764.4 ng/mL, IQR 865.7–4024.3) and PAT (2845.5 ng/mL, IQR 917.8–4719.3). In these locations, over half of the children (50%) had boron levels at or above the 80th percentile (≥ 2543.4 ng/mL) (Table 2).

Both, MAN and CAM, had the highest median levels of Cadmium (MAN: 0.42 ng/mL, IQR 0.24–0.48; CAM: 0.36 ng/mL, IQR 0.28–0.37). However, the proportion of children with high-level exposure to cadmium (≥ 0.40 ng/mL) differed between these locations, with 63.6% ($n$= 7/11) in MAN and 40.0% ($n$= 4/10) in CAM (Table 2). However, this difference was not statically significant ($p$= 0.279).

The results of urine metals and metalloids were adjusted for specific gravity to account for urine dilution and standardize measurements. It is noteworthy that children who self-reported chronic non-communicable diseases and those experiencing chronic illnesses at the time of evaluation were excluded from this study. Additionally, it was confirmed that the urine specific gravity values of the participants fell within the normal range established for girls and boys within the age range of the study participants.

In Mexico, a country with diverse economic and industrial activities and limited research funding, assessing pollutants such as heavy metals and metalloids in large sample sizes is often cost-prohibitive. Therefore, this research represents a significant collaborative effort involving multiple institutions, funded by the federal government, aimed at comprehensively characterizing the health status of children in terms of exposure to the analyzed xenobiotics. By addressing this knowledge gap, the study aims to provide valuable insights into the environmental health challenges faced by children. Another major strength is our ability to corroborate the dispersion of metals and metalloids reported in the RETC.

The potential limitations inherent in a cross-sectional study design should be carefully acknowledged when interpreting our findings. Additionally, our study employed a self-recruitment strategy within schools where eligible children received education. As a result, the sample of girls and boys evaluated may not be fully representative of the source population. We must acknowledge that we were unable to capture the full spectrum of intrinsic and extrinsic factors that may influence both exposure and KIM-1 levels.

Finally, we did not evaluate correlations between the analyzed heavy metals and metalloids. Evaluating these correlations is of paramount importance in understanding their potential impact on children's health. Correlations between these heavy metals can provide insights into potential sources of exposure and pathways of toxicity.

Despite these limitations, our study provides valuable insights into the relationship between heavy metal exposure and renal biomarkers in school-aged children, underscoring the need for further research to confirm and expand upon our findings.

## Conclusions

While our study identifies potential associations between regional pollutant burdens and KIM-1 levels in children, it also underscores the critical need for tailored mitigation strategies informed by the unique pollutant profiles of each region. These profiles should consider specific environmental and industrial activities that contribute to local exposures. Replicating this study in other populations is crucial to assess the generalizability of these findings. Further research is needed to elucidate the mechanisms underlying the observed associations between specific heavy metals and metalloids and KIM-1, irrespective of future replications. Establishing safe exposure levels for this specific population is also required.

## Author contributors

This study was conceptualized and designed by O.M.C. and M.R.S. Data collection involved a collaborative effort by O.M.C., M.R.S., I.E.G.C., A.A.C.d.l.C., and F.R.G. E.M.Z., H.B.C.A., and M.A.M.P. performed the data analysis. Interpretation of the results involved contributions from O.M.C., M.R.S., E.M.Z., H.B.C.A., A.L.Q.M., M.A.M.P., and P.A.R.A. O.M.C. and E.M.Z. wrote the first draft, which was reviewed and edited by M.R.S., I.E.G.C., A.A.C.d.l.C., F.R.G., H.B.C.A., A.L.Q.M., M.A.M.P., P.A.R.A., and A.G.H.L. All authors approved the final version of the manuscript submitted for publication.

### Supplementary Information

Supplementary Information.

## Data Availability

Data requests should be sent to the corresponding author and will be reviewed by lead investigators and the funding council.
