# Supplementary material for: Metal concentrations and KIM-1 levels in school-aged children: a cross-sectional study
Source: Sci Rep. 2024 Jun 12;14:13464. doi: 10.1038/s41598-024-62320-8 (PMC11169506; doi:10.1038/s41598-024-62320-8)
Supplement: Supplementary file 1 — Supplementary Information. [file 41598_2024_62320_MOESM1_ESM.docx]

**Supplementary data**

| **Supplementary data 1.** Characteristics of the study sample for selected variables in 225 girls and boys, Mexico 2023 | | | | | |
| --- | --- | --- | --- | --- | --- |
| **Characteristic** | **Overall** |  | **Girls** | **Boys** | $\boldsymbol{p}$ |
|  | $n$ = 225 |  | $n$ = 115 | $n$ = 110 |  |
| Age (mean ± S.D.), years | 8.2 ± 1.6 |  | 8.3 ± 1.6 | 8.1 ± 1.6 | 0.333 |
| Age group (tertiles), years |  |  |  |  |  |
| 5 – 7 | 83 (36.9) |  | 41 (35.7) | 42 (38.2) | 0.542 |
| 8 – 9 | 92 (40.9) |  | 45 (39.1) | 47 (42.7) |  |
| 10 – 12 | 50 (22.2) |  | 29 (25.2) | 21 (19.1) |  |
| Height (mean ± S.D.), cm. | 131.1 ± 11.5 |  | 131.8 ± 11.8 | 130.3 ± 11.2 | 0.320 |
| Weight (mean ± S.D.), kg. | 31.6 ± 11.1 |  | 32.1 ± 11.9 | 31.1 ± 11.1 | 0.536 |
| Locality of residence |  |  |  |  |  |
| COL | 36 (16.0) |  | 22 (19.1) | 14 (12.7) | 0.512 |
| TEC | 15 (6.7) |  | 7 (6.1) | 8 (7.3) |  |
| ARM | 27 (12.0) |  | 13 (11.3) | 14 (12.7) |  |
| CAL | 16 (7.1) |  | 8 (7.0) | 8 (7.3) |  |
| MAN | 39 (17.3) |  | 22 (19.1) | 17 (15.5) |  |
| CAM | 21 (9.3) |  | 9 (7.8) | 12 (10.9) |  |
| QUE | 54 (24.0) |  | 23 (20.0) | 31 (28.2) |  |
| MIN | 5 (2.2) |  | 2 (1.7) | 3 (2.7) |  |
| PAT | 12 (5.3) |  | 9 (7.8) | 3 (2.7) |  |
| *Nutritional status:* |  |  |  |  |  |
| Weight-for-age |  |  |  |  |  |
| Malnutrition (mild – moderate) | 40 (17.8) |  | 19 (16.5) | 21 (19.1) | 0.103 |
| Normal | 120 (53.3) |  | 69 (60.0) | 51 (46.4) |  |
| Overweight – Obesity | 65 (28.9) |  | 27 (23.5) | 38 (34.5) |  |
| Height-for-age |  |  |  |  |  |
| Low | 32 (14.2) |  | 15 (13.0) | 17 (15.5) | 0.799 |
| Normal | 158 (70.2) |  | 83 (72.2) | 75 (68.1) |  |
| High | 35 (15.6) |  | 17 (14.8) | 18 (16.4) |  |
| BMI-for-age |  |  |  |  |  |
| Low | 8 (3.6) |  | 4 (3.5) | 4 (3.6) | 0.628 |
| Normal | 138 (61.3) |  | 74 (64.3) | 64 (58.2) |  |
| Overweight – Obesity | 79 (35.1) |  | 37 (32.2) | 42 (38.2) |  |
| *Ever medical diagnosis of (any, yes)* |  |  |  |  |  |
| Chronic respiratory disease | 17 (7.6) |  | 7 (6.1) | 10 (9.1) | 0.394 |
| Neurological disease | 17 (7.6) |  | 9 (7.8) | 8 (7.3) | 0.875 |
| Immunological or allergic disease | 30 (13.3) |  | 18 (15.7) | 12 (10.9) | 0.295 |
| Psychiatric disease | 34 (15.1) |  | 14 (12.2) | 20 (18.2) | 0.208 |
| Abbreviations: SD, Standard Deviation; BMI, Body Mass Index; COL, Colima – Villa de Álvarez; TEC, Tecomán; ARM, Armería; CAL, Caleras; MAN, Manzanillo; CAM, Campos; QUE, Quesería; MIN, Minatitlán; PAT, Paticajo.  Notes: 1) The absolute (n) and relative (%) frequencies are presented, unless the arithmetic mean and SD are specified; 2) The p-values from chi-squared or t tests are presented accordingly; 3) The nutritional status was evaluated using Z scores determined with the WHO Child Growth Standards; 4) The following medical diagnoses were considered in the clusters of personal history of diseases: Chronic respiratory disease (asthma, emphysema, chronic bronchitis, or pulmonary fibrosis); Neurological disease (stroke, migraine, epilepsy, neuropathy, or myopathy); Immunological or allergic disease (atopic dermatitis, lupus, rheumatoid arthritis, allergic rhinitis, or allergic conjunctivitis); Psychiatric disease (depression, anxiety, bipolar disorder, eating disorders, attention deficit hyperactivity disorder, or neurodivergent disorder). | | | | | |
